# GEFs: Dual regulation of Rac1 signaling

**DOI:** 10.1080/21541248.2016.1202635

**Published:** 2016-06-17

**Authors:** Hadir Marei, Angeliki Malliri

**Affiliations:** Cell Signaling Group, Cancer Research UK Manchester Institute, The University of Manchester, Manchester, UK

**Keywords:** cell contraction, cell invasion, cell migration, guanine nucleotide exchange factors (GEFs), IQGAP1, MLC; NMMIIA; PRex-1; FLII; Rac1; Tiam1

## Abstract

GEFs play a critical role in regulating Rac1 signaling. They serve as signaling nodes converting upstream signals into downstream Rac1-driven cellular responses. Through associating with membrane-bound Rac1, GEFs facilitate the exchange of GDP for GTP, thereby activating Rac1. As a result, Rac1 undergoes conformational changes that mediate its interaction with downstream effectors, linking Rac1 to a multitude of physiological and pathological processes. Interestingly, there are at least 20 GEFs involved in Rac1 activation, suggesting a more complex role of GEFs in regulating Rac1 signaling apart from promoting the exchange of GDP for GTP. Indeed, accumulating evidence implicates GEFs in directing the specificity of Rac1-driven signaling cascades, although the underlying mechanisms were poorly defined. Recently, through conducting a comparative study, we highlighted the role of 2 Rac-specific GEFs, Tiam1 and P-Rex1, in dictating the biological outcome downstream of Rac1. Importantly, further proteomic analysis uncovered a GEF activity-independent function for both GEFs in modulating the Rac1 interactome, which results in the stimulation of GEF-specific signaling cascades. Here, we provide an overview of our recent findings and discuss the role of GEFs as master regulators of Rac1 signaling with a particular focus on GEF-mediated modulation of cell migration following Rac1 activation.

## Introduction

**Figure 1.**
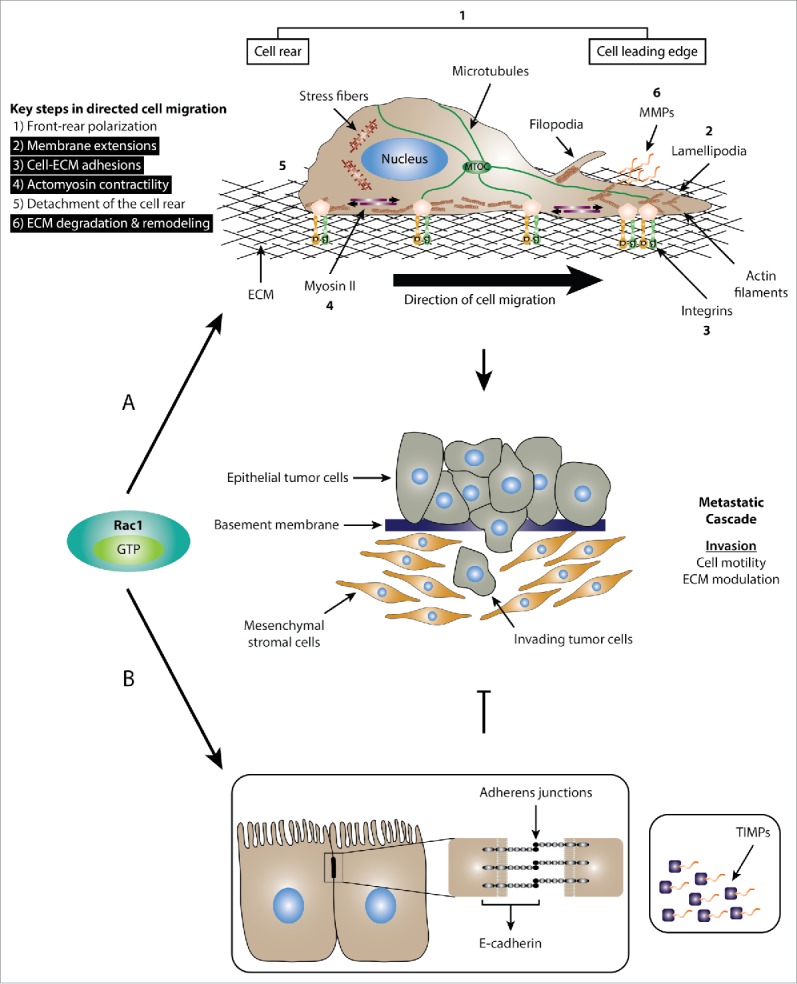
Dual role of Rac1 in cell migration. (A) Schematic representation of the role of Rac1 in promoting cell migration and invasion. Mesenchymal cell migration and invasion is governed by a number of key cellular events, including 1) front-rear polarization characterized by the acquisition of an asymmetrical morphology, reorientation of the nucleus and repositioning of the microtubule organizing center (MTOC) in front of the nucleus; 2) formation of membrane protrusions, including lamellipodia, filopodia and invadopodia; 3) stimulation of focal complex and focal adhesion assembly and turnover; 4) actomyosin contractility to generate the traction force required for cell movement; 5) detachment of the cell rear to allow forward movement of cells; 6) ECM degradation and remodeling through the action of proteases, such as MMPs. Activation of Rac1 is implicated in a number of cellular processes (highlighted in black) that drive cell migration and invasion. (B) Schematic representation of the role of Rac1 in inhibiting cell migration and invasion. Upon activation, Rac1 has been shown to enhance the formation of E-cadherin-mediated cell-cell adherens junctions, which is linked to reduced cell motility and invasion. Additionally, Rac1 also regulates the expression of TIMPs, which counteract the effect of MMPs, thereby inhibiting ECM degradation and reducing cell invasion.

The small guanosine triphosphate phosphohydrolase (GTPase) Ras-related C3 botulinum toxin substrate 1 (Rac1) plays a critical role in regulating cell migration and invasion. Although, this is required for a number of physiological processes, such as embryonic development, immune responses as well as wound healing, aberrant Rac1 signaling and the associated deregulation of cell motility and invasion is a hallmark of cancer metastasis, the leading cause of death in cancer patients.^1-4^ Given the adverse symptoms associated with cancer metastasis as well as the limited treatment options available in advanced cancer stages, efforts are now focused on uncovering effective pharmacological modulators that target cancer metastasis. This promises to increase treatment options and enhance patient survival via confining tumors to the initial site of formation.^5^ Given the importance of Rac1 in modulating cell migration and invasion, 2 key steps in the metastatic cascade,^1,3,6^ Rac1 presents an attractive therapeutic target.^7^ However, research into Rac1-driven cell migration and invasion suggest both inhibitory and stimulatory roles (Fig. 1).^2^ As such, a thorough understanding of the molecular mechanisms involved in dictating Rac1 anti- versus pro-metastatic effects are required to aid the development of effective anti-cancer therapies that spare the anti-metastatic functions of Rac1.

Central to Rac1 signaling are guanine nucleotide exchange factors (GEFs). Similar to other GTPases, Rac1 acts as a molecular switch cycling between an inactive guanosine diphosphate (GDP)-bound state and an active guanosine triphosphate (GTP)-bound state. GEFs serve as Rac1 activators via promoting the dissociation of GDP from Rac1, thereby facilitating GTP binding. The GEF family comprises more than 80 members, with at least 20 GEFs implicated in directly activating Rac1. GEFs are further divided into the Dbl or the DOCK families, which differ in the domain mediating their GEF activity. Dbl GEFs possess a characteristic Dbl homology (DH) domain that is responsible for its GEF activity, while DOCK GEFs lack the DH domain and instead possess 2 highly conserved regions known as DOCK-homology region 1 and 2 (DHR1 and DHR2, respectively), with the DHR2 mediating the GEF activity.^8,9^ Interestingly, deregulation of a number of GEFs has been reported in cancer and is generally associated with tumor progression and poor patient outcome.^4^ This, together with the large number of identified GEFs, hints at a complex role of GEFs in regulating Rac1 signaling, beyond serving as Rac1 activators. Indeed, a number of studies have demonstrated the ability of GEFs to influence Rac1 downstream signaling,^10-15^ yet mechanisms governing this process were poorly defined. Recently, we have described a GEF activity-independent role for 2 Rac-specific GEFs, T-cell lymphoma invasion and metastasis-1 (Tiam1) and phosphatidylinositol-3, 4, 5-trisphosphate-dependent Rac exchange factor 1 (P-Rex1), in the modulation of the Rac1 interactome. This, in turn, allows the stimulation of GEF-specific signaling cascades that determine whether Rac1 inhibits or promotes cell migration.^16,17^ Here, we outline our recent findings and discuss the different mechanisms by which GEFs regulate Rac1 signaling, particularly in governing Rac1-driven cell migration.

## Dual role of Rac1 in cell migration and invasion

Cell migration and invasion of the surrounding extracellular matrix (ECM) is a highly complex process with multiple steps that involve alterations in the actin cytoskeleton, cell-cell adhesions, cell-ECM interactions and cell contractile forces.^6,18^ In general, cell migration can be classified into single cell migration including mesenchymal or amoeboid motility, and collective cell migration in the form of cell sheets, strands, tubes, or clusters.^6,19^ Activation of Rac1 has been heavily linked to modulating mesenchymal cell motility.^6^

A hallmark of mesenchymal motility is a morphological change that includes the adoption of a mesenchymal-like morphology with a distinguished cell leading edge and a lagging tail that dictate the directionality of migration. An important consequence of the asymmetrical cellular phenotype is the formation of membrane extensions, including lamellipodia and filopodia. These protrusions help in sensing the environment as well as regulating cell-ECM adhesions at the leading edge that provide the traction force required to propel the cell forward. Actomyosin contractility also contributes to force generation, facilitating cell movement and tail detachment.^6,18^ Mesenchymal motility also involves the degradation of the ECM through the action of secreted proteases, such as matrix metalloproteinases (MMPs).^20^

Evidence from *in vitro* and *in vivo* studies support a role of Rac1 in a number of cellular events governing mesenchymal cell motility (Fig. 1A). Of particular importance is the role of Rac1 in lamellipodia formation through promoting branched actin polymerization at the leading edge. Upon activation, Rac1 associates with its downstream effector insulin receptor tyrosine kinase substrate p53 (IRSp53), which promotes Rac1 binding to WASP-family verprolin-homologous (WAVE) proteins. This leads to conformational changes in the WAVE proteins exposing their C-terminal verprolin-like, central and acidic (VCA) domain, which mediates the binding to and activation of the actin nucleating protein, actin-related protein 2/3 (Arp2/3) protein complex. As a consequence, branched actin polymerization is enhanced at the leading edge, thereby supporting lamellipodia formation.^21^ Via promoting actin-rich protrusions, Rac1, together with the closely related small GTPase cell division control protein 42 homolog (Cdc42), also regulate the formation and turnover of small integrin-dependent focal complexes and adhesions that attach the protrusions to the ECM, thus generating necessary pulling forces that mediate cell migration.^2,22,23^ Additionally, Rac1 has also been shown to regulate the expression of various MMPs, which are required for the proteolytic degradation of the ECM.^6^

Intriguingly, despite the role of Rac1 in promoting cell migration and invasion, activation of Rac1 can also hinder cell migration (Fig. 1B). For example, Rac1 and the Rac-specific GEF, Tiam1, play a critical role in regulating cadherin-mediated cell-cell adhesions,^24-27^ destabilization of which is a prerequisite for single cell motility.^28^ Consistently, it has been shown that phosphorylation of Tiam1 by the oncoprotein sarcoma-family kinase (Src) and its subsequent degradation, specifically at adherens junctions, is required for optimal Src-mediated adherens junction disassembly and enhanced cell migration.^29^ The antagonizing role of Tiam1-Rac1 signaling also extends to growth factor-stimulated cell scattering and invasion. For example, proteasomal degradation of Tiam1, predominantly from cell-cell adhesions, is essential for efficient hepatocyte growth factor (HGF)-induced cell scattering and invasion in various cell lines.^30^ Knockdown of Tiam1 *in vitro* has also been shown to increase cell migration in Madin-Darby canine kidney II (MDCKII) cells due to weaker cadherin-mediated cell-cell contacts.^31^ In addition to their role in regulating the integrity of cell junctions, Tiam1 and Rac1 were also shown to impede cell invasion via protecting against ECM degradation through upregulating tissue inhibitor of metalloproteinase-1 (TIMP-1) and -2 (TIMP-2) while not affecting the secreted levels or activity of MMP-9 or MMP-2.^26^ Together, these studies highlight the dual role of Rac1 signaling in regulating cell migration and invasion. Thus, targeting Rac1 in a clinical setting might, in fact, lead to adverse effects. This calls for the identification of factors that dictate the biological outcome downstream of Rac1 activation to specifically target Rac1-driven signaling cascades that promote cell migration and invasion.

## GEFs as master regulators of Rac1 signaling

### 

#### GEFs dictate the role of Rac1 in cell migration

In our efforts to better characterize the underlying mechanisms involved in regulating Rac1-driven cellular processes, we set out to identify factors that influence the outcome of Rac1 activation on cell migration.

The dual role of Rac1 signaling in regulating cell migration and invasion is often attributed to differences in the cell type in which Rac1 is activated as well as the extracellular signaling inputs.^32^ However, given the large number of GEFs associated with activating Rac1,^8^ together with evidence supporting a role of GEFs in dictating Rac1 downstream signaling,^10-15^ we were interested in examining whether activation of Rac1 by different GEFs can also contribute to its dual role in migration and invasion. Intriguingly, we found that activation of Rac1 by 2 Rac-specific GEFs, Tiam1 and P-Rex1, was associated with differential Rac1-driven cellular effects. Expression of Tiam1 wild type (WT), but not a GEF-dead (GEF*) mutant, enhanced actin and E-cadherin localization at cell-cell contacts, thereby promoting cellular aggregation and the formation of an epithelial-like morphology concomitant with a significant reduction in cell migration. In sharp contrast, P-Rex1 WT expression was associated with cell-cell contact dissociation, formation of elongated actin-rich membrane protrusions and a marked increase in cell migration. All together, these data highlight the importance of Tiam1 and P-Rex1, not only in activating Rac1, but also in dictating Rac1-driven biological outcomes that govern cell migration and invasion.^16,17^

#### GEF-mediated regulation of the Rac1 interactome

Further characterization of the role of Tiam1 and P-Rex1 in dictating Rac1 downstream signaling also uncovered a GEF activity-independent function for both GEFs in modulating the Rac1 interactome. Through conducting a comparative quantitative proteomic analysis, we identified a subset of Rac1 interactors that showed changes in Rac1 binding upon expression of either GEF, highlighting a role for Tiam1 and P-Rex1 in differentially regulating Rac1-protein interactions. Interestingly, bioinformatics analysis of the identified proteins indicated that, via modulating the binding affinity of Rac1 interactors, Tiam1 and P-Rex1 stimulate GEF-specific signaling cascades that could account for the opposing Rac1-driven migratory effects.^16,17^

The underlying mechanism governing the GEF-mediated regulation of the Rac1 interactome was further unraveled through the identification of the gelsolin protein superfamily member, protein flightless-1 homolog (FLII), as a novel Rac1 interactor. Interestingly, FLII displayed a significant increase in Rac1 binding upon expression of P-Rex1 WT but not Tiam1 WT or the GEF* mutants. Importantly, although additional data we obtained indicated that FLII binds preferentially to active Rac1, Tiam1-mediated Rac1 activation did not enhance Rac1-FLII association, highlighting an additional level of regulation beyond Rac1 activation. Further biochemical analysis also revealed that FLII binds to P-Rex1, but not Tiam1, through the FLII gelsolin (GEL) domain, while Rac1 interacts with the leucine rich repeats (LRR) domain of FLII. Therefore, our data indicate that P-Rex1, in addition to activating Rac1, also serves as a scaffolding protein that stimulates active Rac1 binding to the FLII LRR domain, through directly binding to the FLII GEL domain itself. It is unclear, however, whether a transient P-Rex1-Rac1-FLII ternary complex is formed in the process, or whether FLII is transferred from P-Rex1 to Rac1 in a sequential manner. Intriguingly, the P-Rex1-FLII interaction was not dependent on P-Rex1 GEF activity. Indeed, despite the inability of the P-Rex1 GEF* mutant to stimulate the Rac1-FLII interaction, the mutant displayed similar binding to FLII as P-Rex1 WT, highlighting a GEF activity-independent scaffolding role of P-Rex1.^16,17^

**Figure 2.**
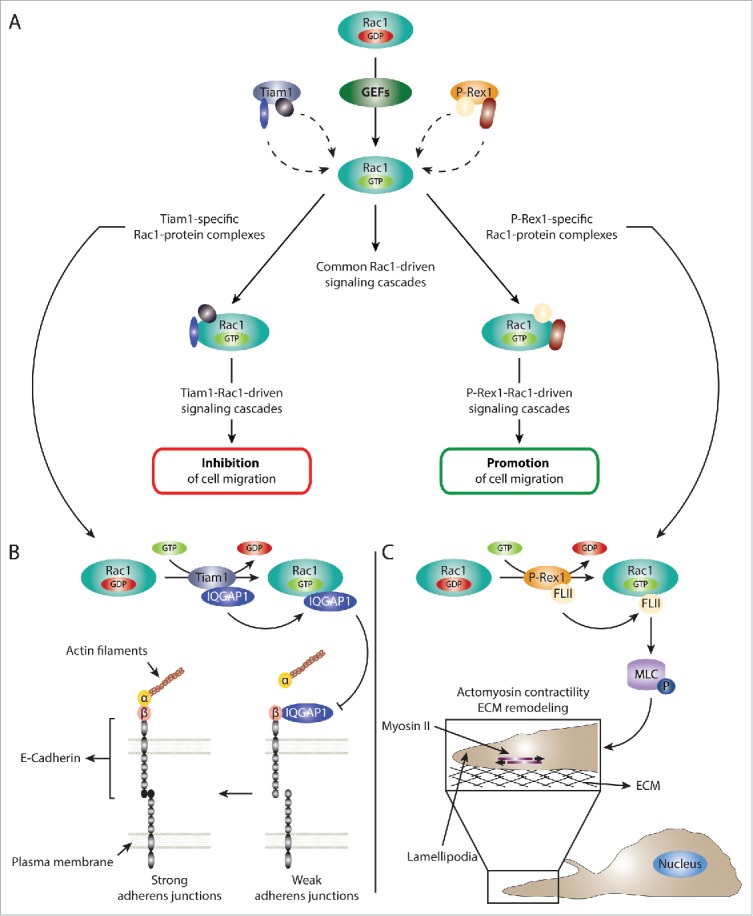
GEFs regulate Rac1 signaling via serving as Rac1 activators as well as scaffolding proteins. (A) Schematic representation of the dual role of GEFs in regulating Rac1 signaling. GEFs activate Rac1 via facilitating the exchange of GDP for GTP. This leads to the association of Rac1 to downstream effectors and the stimulation of various downstream signaling cascades. In addition to acting as Rac1 activators, GEFs can also serve as scaffolding proteins. For example, our data together with evidence from the literature suggest that the scaffolding role of Tiam1 and P-Rex1, 2 Rac-specific GEFs, is important for differentially modulating the Rac1 interactome. As a result, activation of Rac1 by either GEF mediates the formation of a number of GEF-specific Rac1-protein complexes that are important for dictating GEF-specific Rac1 downstream signaling cascades. For simplicity, multiple GEF-specific Rac1-protein interactors are depicted on the same Rac1 molecule; however, it is likely that each GEF drives multiple spatially and temporally distinct Rac1-protein complexes. (B) Schematic representation of the proposed Tiam1-Rac1-IQGAP1 signaling cascade leading to reduced cell migration. Tiam1-mediated Rac1 activation enhances Rac1 binding to IQGAP1. Based on information from the literature, this, in turn, can reduce IQGAP1-β-catenin binding, allowing the formation of stable α-catenin-β-catenin-E-cadherin complexes, thereby leading to stronger adherens junctions. Thus, negative regulation of IQGAP1-mediated cell-cell contact dissociation, might explain the stronger E-cadherin-mediated junctions and reduced cell migration associated with Tiam1-mediated Rac1 activation. (C) Schematic representation of the P-Rex1-Rac1-FLII signaling cascade leading to enhanced cell migration. Through serving as a Rac1 GEF as well as a scaffolding protein, P-Rex1 activates Rac1 while enhancing its interaction with FLII. This leads to increased phosphorylation (depicted by P) of MLC and the activation of myosin II. Given the colocalization of P-Rex1, FLII and actin in lamellipodia, the stimulation of myosin II leads to increased actomyosin contractility and ECM remodeling, potentially at the leading edge. Together, this promotes Rac1-driven cell migration.

A scaffolding function has also been described for Tiam1. For example, it has been shown that Tiam1 can bind directly to Rac1 effectors, such as IRSp53, as well as to scaffolding proteins, including JNK interacting protein 2 (IB2/JIP2) and spinophilin, allowing Tiam1 to stimulate specific Rac1-driven signaling cascades.^11-14^ Interestingly, our proteomic analysis highlighted a number of Tiam1-enriched Rac1 binding partners.^16,17^ Given the importance of the P-Rex1 scaffolding function in stimulating P-Rex1-specific Rac1 interactions, together with the previously reported scaffolding role of Tiam1, it is likely that the observed Tiam1-mediated modulation of the Rac1 interactome is also a consequence of Tiam1 serving as a scaffolding protein. Indeed, both our proteomic screen and further biochemical validation identified the known Rac1 interactor, Ras GTPase-activating-like protein 1 (IQGAP1), as a Tiam1-enriched Rac1 binding partner.^16,17^ Interestingly, in a recent proteomic study, both Tiam1 and Rac1 were shown to interact with IQGAP1,^33^ further supporting the notion that Tiam1 modulates the Rac1 interactome via its scaffolding function. All together, this suggests that GEFs possess 2 separate, yet interdependent functions: 1) the typical GEF function responsible for Rac1 activation via promoting the exchange of GDP for GTP and 2) a GEF activity-independent scaffolding role that regulates the nature of Rac1-protein interactions. However, both functions are required in order to stimulate GEF-specific Rac1 downstream signaling cascades (Fig. 2A).

#### GEF-mediated signaling cascades governing Rac1-driven cell migration

Similar to other gelsolin protein superfamily members, FLII plays an essential role in regulating actin dynamics via barbed-end actin capping, actin severing and regulation of other actin binding proteins, such as diaphanous-related formins (DRFs).^34-36^ As such, FLII has been implicated in regulating cell migration. Indeed, FLII was shown to translocate from the nucleus to membrane ruffles and lamellipodia at the leading edge in motile cells where it colocalizes with Ras and small GTPases, including Ras homolog gene family member A (RhoA) and Cdc42.^37^ Interestingly, although a number of studies highlight a negative role of FLII in wound healing and cell migration,^36,38-42^ fibroblasts derived from FLII heterozygous mice exhibit reduced contractile abilities compared to their WT counterparts.^42^ This indicates that, in addition to regulating actin dynamics, FLII is also implicated in mediating cell contraction, a key step in driving mesenchymal cell motility,^6,18^ thus highlighting a potential dual role of FLII in regulating cell migration.

Central to cell contraction is the activation of myosin II through phosphorylation of myosin light chain (MLC). Indeed, MLC phosphorylation mediated by RhoA and its downstream effector Rho-associated serine/threonine kinase (ROCK) promotes stress fiber formation and focal adhesion assembly in the cell center of migrating cells, thereby mediating actomyosin contractility.^43^ Additionally, a separate pool of phosphorylated MLC has also been described at the leading edge of motile cells.^21,44^ Interestingly, FLII was shown to regulate the activation of nonmuscle myosin IIA (NMMIIA) at cell protrusions to promote the formation of cell extensions that mediate collagen remodeling.^45^ This suggests that FLII might regulate cell contraction at the leading edge in a RhoA-ROCK-independent manner.

Given the P-Rex1-medaited stimulation of the Rac1-FLII interaction concomitant with enhanced cell migration, we further investigated the role of FLII in Rac1-driven cell migration. Our results indicated that FLII is required for optimal cell migration, supporting a dual role of FLII in regulating cell motility. Importantly, depletion of FLII completely abrogated P-Rex1s ability to stimulate Rac1-driven cell migration, demonstrating the importance of FLII in this process. Interestingly, additional functional analysis revealed a novel FLII-dependent role of P-Rex1 in regulating MLC phosphorylation and cell contraction, which was not observed with Tiam1. P-Rex1-mediated cell contraction was also associated with increased collagen crosslinking and remodeling.^16^ Additionally, consistent with the reported role of FLII in modulating NMMIIA activation at cell protrusions to regulate collagen remodeling,^45^ we found that both FLII and P-Rex1 colocalize at the leading edge of migrating cells.^16^ Together, these data suggest that P-Rex1 regulates cell contraction, likely at the leading edge, in a FLII-dependent manner to mediate Rac1-driven cell migration. Indeed, inhibition of actomyosin contractility using blebbistatin reduced P-Rex1-Rac1-driven cell migration. Importantly, inhibition of RhoA-ROCK-dependent MLC phosphorylation and cell contraction using the ROCK inhibitor Y27632 had no effect on P-Rex1-Rac1-driven cell migration.^16^ This indicates that P-Rex1, through FLII, regulates the activation of a separate pool of MLC than that regulated by RhoA-ROCK signaling, potentially at the leading edge. Our data also highlights a role of these P-Rex1-FLII-mediated contractile forces in ECM remodeling, which might be particularly important in cancer cell dissemination (Fig. 2).

In addition to its role in regulating cell contraction, FLII has also been reported to regulate the activity of DRFs, such as diaphanous-related formin-1 (mDia1).^35^ Although, mDia1 is a Rho effector, it has also been linked to Rac1 activation and the formation of membrane ruffles. Additionally, mDia1 is also implicated in focal adhesion remodeling and migration of cancer cells.^46^ Therefore, it is possible that the Rac1-FLII association plays a role in enhancing DRFs' activity to promote cell migration. It would be informative to look at the ability of FLII to bind to and activate DRFs, such as mDia1, in response to P-Rex1 expression or deletion. This might uncover yet another novel role of P-Rex1 in regulating Rac1-driven cellular effects.

In contrast to P-Rex1, Tiam1-mediated activation of Rac1 was associated with reduced cell migration.^16^ Functional validation of Tiam1-enriched Rac1 interactors identified from our proteomic screen indicated that Tiam1-mediated activation of Rac1 enhances Rac1-IQGAP1 binding.^17^ Intriguingly, IQGAP1 was shown to disrupt cell-cell contacts via binding to β-catenin and destabilizing the α-catenin-β-catenin-E-cadherin complex.^47^ However, increased binding of active Rac1 to IQGAP1 inhibits its binding to β-catenin, thereby stabilizing E-cadherin-mediated cell-cell contacts.^48^ Together, this suggests that the observed reduction in cell migration associated with Tiam1-mediated Rac1 activation might be a consequence of stronger cell-cell contacts through inhibiting IQGAP1 binding to β-catenin. Consistently, expression of Tiam1 WT, but not P-Rex1 WT or the GEF* mutants, enhanced E-cadherin localization at cell-cell contacts contaminant with increased cellular aggregation.^16,17^ Additionally, Tiam1-mediated activation of Rac1 is also associated with reduced HGF- and epidermal growth factor (EGF)-induced cell scattering, further demonstrating the importance of Tiam1-Rac1 signaling in stabilizing cell-cell contacts.^16^ Thus, the modulation of the Rac1-IQGAP1 interaction highlights a potential role of Tiam1, not only in stimulating specific signaling cascades, but also in negatively regulating other protein-protein interactions through directing activated Rac1 to pre-existing complexes in the cell, ultimately leading to reduced cell migration (Fig. 2).

## Conclusions and future perspectives

Our recent findings provide direct evidence implicating GEFs in modulating Rac1 downstream signaling via regulating the Rac1 interactome, a function that is likely mediated through the scaffolding role of GEFs. Indeed, through promoting GEF-specific Rac1-protein interactions, we show that Tiam1 and P-Rex1 determine whether Rac1 will inhibit or promote cell migration, respectively (Fig. 2). Additionally, our data indicate that the scaffolding function of GEFs is independent of their GEF activity, yet Rac1 activation is still required for effector binding. This warrants additional examination of the interdependency between these 2 GEF functions, as the scaffolding role might represent a potentially important regulatory mechanism that could influence cancer dissemination promoted by Rac1 activating mutations.^4^ Given the large number of GEFs identified to date, together with their differential tissue expression patterns and the diverse upstream signals involved in their activation,^8,9^ it would also be interesting to adopt a comparative proteomic approach, similar to that utilized for Tiam1 and P-Rex1, using other GEFs.^16,17^ This will help further elucidate the importance of the GEF scaffolding function in regulating Rac1 signaling under different physiological conditions. Moreover, it promises to highlight additional GEF-specific Rac1-protein complexes that will aid in a better understanding of the contrasting role of Rac1 in cellular events, such as migration and invasion.

The highlighted data also represents a significant step-up in our understanding of how P-Rex1 mediates Rac1-driven cell migration in both normal and cancer cells. This is particularly important, since upregulation of P-Rex1 has been implicated in promoting metastasis in prostate cancer as well as in melanoma.^49,50^ Additionally, P-Rex1 has also been identified as an important mediator of erythroblastic leukemia viral oncogene homolog (ErbB) receptor signaling, promoting Rac1-driven migration and proliferation in breast cancer.^51^ Moreover, elevated P-Rex1 expression has been associated with poor patient outcome in breast cancer.^52^ Interestingly, a novel Rac1 inhibitor, 1A-116, which interferes with Rac1-P-Rex1 binding and suppresses Rac1 activation, was shown to efficiently diminish the formation of metastatic lung colonies in a breast cancer metastasis mouse model.^53^ While the selectivity of this compound towards specifically targeting Rac1-P-Rex1 binding versus other Rac1-GEF complexes is still under investigation, nevertheless, these preclinical data highlight the therapeutic potential of targeting PRex1-specific Rac1 signaling cascades as an effective anti-cancer therapy. Therefore, highlighting the scaffolding role of P-Rex1 and uncovering the signaling cascade governing its role in regulating MLC phosphorylation, cell contraction and ECM remodeling might, in fact, aid in the development of more effective Rac1 inhibitors that spare Rac1's physiological and anti-metastatic tumor functions.

## Abbreviations

Arp2/3: Actin-related protein 2/3
Cdc42: Cell division control protein 42 homolog
DH: Dbl homology
DHR: DOCK-homology region
DRF: Diaphanous-related formin
ECM: Extracellular matrix
EGF: Epidermal growth factor
ErbB: Erythroblastic leukemia viral oncogene homolog
FLII: Protein flightless-1 Homolog
GDP: Guanosine diphosphate
GEF: Guanine nucleotide exchange factor
GEF*: GEF-dead mutant
GEL: Gelsolin-like domain
GTP: Guanosine triphosphate
GTPase: Guanosine triphosphate phosphohydrolase
HGF: Hepatocyte growth factor
IB2/JIP2: JNK interacting protein 2
IQGAP1: Ras GTPase-activating-like protein 1
IRSp53: Insulin receptor tyrosine kinase substrate p53
LRR: Leucine rich repeats
MDCKII: Madin-Darby canine kidney II
mDia1: Diaphanous-related formin-1
MLC: Myosin light chain
MMP: Matrix metalloproteinase
MTOC: Microtubule organizing center
NMMIIA: Nonmuscle myosin IIA
P: Phosphate group
P-Rex1: Phosphatidylinositol-3, 4, 5-trisphosphate-dependent Rac exchange factor 1
Rac1: Ras-related C3 botulinum toxin substrate 1
RhoA: Ras homolog gene family member A
ROCK: Rho-associated serine/threonine kinase
Src: Sarcoma-family kinase
Tiam1: T-cell lymphoma invasion and metastasis-1
TIMP: Tissue inhibitor of metalloproteinase
WAVE: WASP-family verprolin-homologous
WT: Wild type
VCA: Verprolin-like, central and acidic
